# Treatment response lowers tumor symptom burden in recurrent and/or metastatic head and neck cancer

**DOI:** 10.1186/s12885-020-07440-w

**Published:** 2020-09-29

**Authors:** Markus Hecht, Dennis Hahn, Philipp Wolber, Matthias G. Hautmann, Dietmar Reichert, Steffi Weniger, Claus Belka, Tobias Bergmann, Thomas Göhler, Manfred Welslau, Christina Große-Thie, Orlando Guntinas-Lichius, Jens von der Grün, Panagiotis Balermpas, Katrin Orlowski, Diethelm Messinger, Karsten G. Stenzel, Rainer Fietkau

**Affiliations:** 1 Department of Radiation Oncology, University Hospital Erlangen, Friedrich-Alexander-Universität Erlangen-Nürnberg, Universitätsstraße 27, 91054 Erlangen, Germany; 2 Klinikum Stuttgart, Klinik für Onkologie, Stuttgart, Germany; 3 Universitätsklinikum Köln, Klinik für Hals-, Nasen- und Ohrenheilkunde, Köln, Germany; 4 Universitätsklinikum Regensburg, Klinik und Poliklinik für Strahlentherapie, Regensburg, Germany; 5 Medizinische Studiengesellschaft NORD-WEST GmbH, Westerstede, Germany; 6 Gemeinschaftspraxis Dres. Weniger/Bittrich, Erfurt, Germany; 7 Klinikum der Universität München (A.ö.R.), Klinik für Strahlentherapie und Radioonkologie, Munich, Germany; 8 SRH Wald-Klinikum Gera GmbH, II. Medizinische Klinik, Gera, Germany; 9 Onkozentrum Dresden/Freiberg, Dresden, Germany; 10 Klinikum Aschaffenburg, Hämato-Onkologische Schwerpunktpraxis, Aschaffenburg, Germany; 11 Universitätsmedizin Rostock, Zentrum Innere Medizin Klinik III – Hämatologie, Onkologie, Palliativmedizin, Rostock, Germany; 12 Universitätsklinikum Jena, Klinik für Hals-Nasen- und Ohrenheilkunde, Jena, Germany; 13 Klinikum der J.-W. Goethe-Universität Frankfurt a.M., Klinik für Strahlentherapie und Onkologie, Frankfurt, Germany; 14 Merck Serono GmbH, Medical Affairs Oncology, Darmstadt, Germany; 15 Prometris GmbH, Mannheim, Germany

**Keywords:** Symptom, Response, HNSCC, Cetuximab, Chemotherapy

## Abstract

**Background:**

Head and neck squamous cell cancer (HNSCC) frequently causes severe symptoms that may be reduced, when the tumor is successfully treated. The SOCCER trial studied the association of treatment response with patient reported tumor symptom burden in first line treatment of recurrent and/or metastatic HNSCC.

**Methods:**

In this prospective, multi-center, non-interventional trial patients were treated either with platinum-based chemotherapy and cetuximab or radiotherapy and cetuximab. Tumor symptom burden was assessed every four weeks with a questionnaire containing ten visual analogue scales (VAS, range 0–100), which were summarized to the overall VAS score.

**Results:**

Fourhundred seventy patients were registered in 97 German centers. A total of 315 patients with at least the baseline and one subsequent questionnaire were available for analysis. Changes in the VAS score were rated as absolute differences from baseline. Negative values indicate improvement of symptoms. The overall VAS score improved significantly at the first post-baseline assessment in responders (− 2.13 vs. non-responders + 1.15, *p* = 0.048), and even more for the best post-baseline assessment (− 7.82 vs. non-responders − 1.97, *p* = 0.0005). The VAS for pain (− 16.37 vs. non-responders − 8.89, *p* = 0.001) and swallowing of solid food (− 16.67 vs. non-responders − 5.06, *p* = 0.002) improved significantly more in responders (best post-baseline assessment). In the multivariable Cox regression analysis, worse overall VAS scores were associated with worse overall survival (hazard ratio for death 1.12 per 10 points increment on the overall VAS scale, 95% CI 1.05–1.20, *p* = 0.0009).

**Conclusion:**

In unselected patients beyond randomized controlled trials, treatment response lowers tumor symptom burden in recurrent and/or metastatic HNSCC.

**Trial registration:**

ClinicalTrials.gov, NCT00122460. Registered 22 Juli 2005,

## Background

Patients with recurrent and/or metastatic head and neck squamous cell carcinoma (HNSCC) still have a poor prognosis. In the last decade many of these patients were treated with palliative chemotherapy with platinum, 5-flurouracil and cetuximab (EXTREME regimen) [1]. Salvage surgery or re-irradiation is a treatment option for selected patients with locoregional recurrences. Re-irradiation can either be administered either alone or in combination with chemotherapy or cetuximab [2, 3]. In recent years immune checkpoint inhibitors targeting the programmed cell death 1 protein (PD-1) / programmed cell death ligand 1 (PD-L1) pathway have become a new treatment option either alone or in combination with chemotherapy [4]. Compared to other tumors, recurrent and/or metastatic HNSCC patients frequently suffer from severe tumor symptom burden such as swallowing problems and pain [5]. In these patients, good palliative treatment should not only focus on survival prolongation, but also to improve tumor symptoms and subsequently improve the patient’s quality of life.

In the SOCCER trial patients with recurrent and/or metastatic HNSCC were treated either with chemotherapy and cetuximab or radiotherapy and cetuximab. The hypothesis of the trial was that this treatment will lead to a better control of tumor-related symptom burden in responding patients. The non-interventional trial focused on the patients’ self-reported tumor symptom burden in treatment responders compared to non-responders. This may support the use of treatment schemes with high response rates for patients with severe tumor symptom burden in future treatment algorithms.

## Methods

### Patients

Patients with recurrent and/or metastatic squamous cell carcinoma of the oral cavity, oropharynx, hypopharynx and larynx were eligible for this study. Key criteria for eligibility were first line systemic treatment in the recurrent and/or metastatic situation and the willingness of the patients to fill in the tumor symptoms questionnaire. As the trial should represent unselected patients, there were no limitations regarding baseline ECOG performance status or blood parameters. Tumor stages were evaluated according to TNM 7th edition.

### Trial design and treatments

In this prospective, multi-center, non-interventional study, patients were treated either with platinum-based chemotherapy in combination with cetuximab or radiotherapy in combination with cetuximab. The treatment with cetuximab was according to the European Medicines Agency (EMA) marketing authorization. Cetuximab was administered at an initial dose of 400 mg per square meter body surface area, followed by subsequent doses of 250 mg per square meter body surface area. In combination with radiotherapy, cetuximab treatment started one week prior to radiotherapy and lasted till the end of radiotherapy. In combination with platinum-based chemotherapy, cetuximab was given concomitantly with chemotherapy and continued as maintenance therapy until disease progression. The allocation to the treatment method was made by the treating physician.

### Endpoints and assessments

The primary endpoint of the trial was the association between the patients’ tumor symptom burden and treatment response. The patients’ tumor symptom burden was studied using a 10 item containing questionnaire. Patients filled in this questionnaire before and every fourth week during treatment. The questionnaire included self-evaluation of pain, breathing, swallowing (solid, mashed, fluid), speech, smelling, taste, physical activity and overall health state. The patients reported the severity of problems on a visual analogue scale (VAS) from 0 till 100 (supplementary Fig. S1). Higher values represent heavier symptoms. The overall VAS score is the average value of the 10 single VAS scores. The patients’ tumor symptom burden should be evaluated every four weeks. For tumor response assessment, RECIST criteria version 1.1 were recommended. There was no central RECIST evaluation. Best overall response (BOR) categories during treatment were determined for each patient as: complete response (CR), partial response (PR), stable disease (SD), progressive diseases (PD) and not assessable (NA). The overall response rate (ORR) was the proportion of patients with CR or PR and the disease control rate (DCR) was the proportion of patients with CR, PR or SD. Secondary endpoints of the trial included overall survival (OS) and progression-free survival (PFS).

### Trial oversight

The trial was registered with ClinicalTrials.gov (identifier: NCT00122460). The institutional review board at the Friedrich-Alexander-Universität Erlangen-Nürnberg (number: 84_12 B) approved the non-interventional trial. All patients gave written informed consent before enrollment. The academic authors designed the trial in collaboration with the sponsor (Merck Serono GmbH).

### Statistical analysis

Target Analysis Set (TAS) was defined as all registered patients who fulfilled the eligibility criteria. The modified TAS (mTAS) was defined as all patients in the TAS, who had at least one evaluable pair of questionnaires before and during treatment. Analysis of covariance (ANCOVA) were used to investigate the association between tumor response (CR/PR vs SD/PD/NA) and the changes in VAS scores from baseline to three time points (1) the first post-baseline assessment, (2) the best post-baseline VAS value and (3) the VAS assessment at treatment end. The baseline VAS values were considered as covariates in the ANCOVA and Least Square Means (LSM) including 95% confidence intervals (CI) were determined.

The Wilson Score method was used to determine 95%CI for ORR and DCR rates. Kaplan-Meier estimates were applied for all time-to-event variables (PFS and OS) to estimates the survival probabilities at various time points and the median survival time after start of treatment.

Cox proportional hazard methods were used to investigate the association between various baseline factors (including the overall VAS symptom score) and OS. A backward selection procedure was applied considering all factor with an effect *p*-value of < 0.2 in the univariate analysis to identify independent prognostic factor for OS. The p-value in the backward selection to remain in the final model was 0.05. SAS version 9.3 was used to perform the statistical analysis.

## Results

### Patients and treatment

**Fig. 1:**
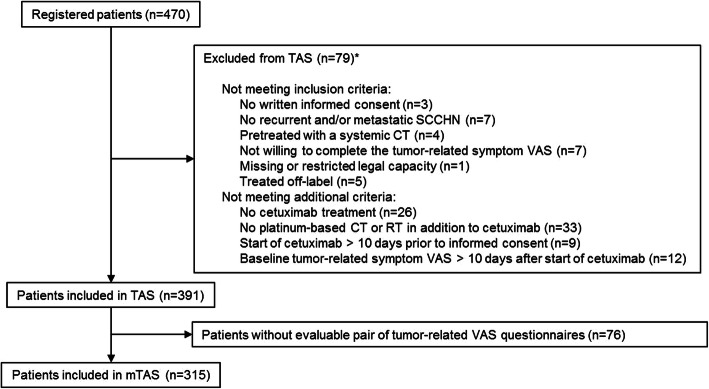
Consort diagram. TAS: Target Analysis Set; mTAS: modified TAS; CT: Chemotherapy; RT: Radiotherapy; VAS: Visual Analogue Scale. *Some patients violated more than one criterion

Between October 2012 and June 2019 a total of 470 patients were registered in 97 German centers. Seventy-nine patients were excluded as they violated at least one eligibility criteria (Fig. 1). The most frequent eligibility criteria violation was a missing combination of cetuximab with radiotherapy or platinum-based chemotherapy. The remaining 391 patients were included in the TAS. Seventy-six patients provided no evaluable pair of questionnaires before and during therapy, and thus in the mTAS 315 patients were evaluable. Clinical characteristics of the 391 TAS patients are given in Table 1. One hundred ninety-eight patients presented with local recurrence only (50.6%), 119 patients had distant metastases only (30%) and 74 patients (19%) had local relapse and distant metastases. Seventy-seven patients with an ECOG score of ≥2 were included (20%) and 124 patients had a Charlson comorbidity score greater than one (32%). Treatment consisted of cetuximab plus radiotherapy in 78 patients (20%) and cetuximab plus chemotherapy in 309 patients (79.0%), 4 patients received both (1%). The chemotherapy was cisplatin based in 174 patients (56%) and carboplatin based in 139 patients (44%). Two hundred sixty-four patients had received prior surgery (68%) and 323 patients prior radiotherapy (83%).

**Table 1:** Patient characteristics

Patient characteristics (TAS cohort, ***N = 391***)	
**Age at study inclusion** [years], median (range)	62 (29–89)
**Weight** [kg], median (range)	66.8 (37–145)
**Sex**, n (%)	
Female	71 (18)
Male	320 (82)
**Location of primary tumor**^a^, n (%)	
Oropharynx	110 (28)
Hypopharynx	94 (24)
Larynx	54 (14)
Oral cavity	97 (25)
Other location	72 (18)
**Prior therapy**, n (%)	
Radiotherapy	320 (82)
Surgery	264 (68)
**Disease progression at study inclusion**, n (%)	
Local recurrence only	198 (51)
Distant metastases only	119 (30)
Local recurrence and distant metastases	74 (19)
**Charlson Comorbidity Index at study inclusion**, n (%)	
0	188 (48)
1	79 (20)
> 1	124 (32)
**ECOG performance status at treatment initiation**, n (%)	
0	65 (17)
1	225 (58)
≥ 2	77 (20)
Missing	24 (6)
**Alcohol consumption**, n (%)	
Never	87 (22)
Several times per month	85 (22)
Several times per week or daily	101 (26)
Missing	118 (30)
**Smoking habits**, n (%)	
Never smoked	102 (26)
Former smoker	148 (38)
Current smoker	140 (36)
Missing	1 (0)
**Pack years**, former and current smoker (*n* = 288), median (range)	35 (1–200)
**Applied treatment regimen**, n (%)	
Radiotherapy + cetuximab	78 (20)
Chemotherapy + cetuximab	309 (79)
Radio-chemotherapy + cetuximab	4 (1)
**Applied chemotherapy regimen** (*n* = 313), n (%)	
Cisplatin-based	174 (56)
Carboplatin-based	139 (44)

*TAS* Target Analysis Set, *ECOG* Eastern Cooperative Oncology Group. ^a^Multiple locations per patient possible

### Response to treatment

The mean follow-up time was 8.6 months (range: 0–33.8). The ORR in the entire cohort was 33% (95%CI: 28.8–38.1) and DCR was 56% (95%CI: 51.3–61.1). In the subgroup chemotherapy-cetuximab the ORR was 32% and in the subgroup radiotherapy-cetuximab 39%. In addition, Kaplan-Meier analyses of OS and PFS were performed in the entire cohort (TAS cohort) (supplementary Fig. S2). The median PFS was 5.5 months (95%CI: 4.8–6.0) and the median OS was 9.5 months (95%CI: 8.5–10.9).

### Baseline symptom burden

**Fig. 2:**
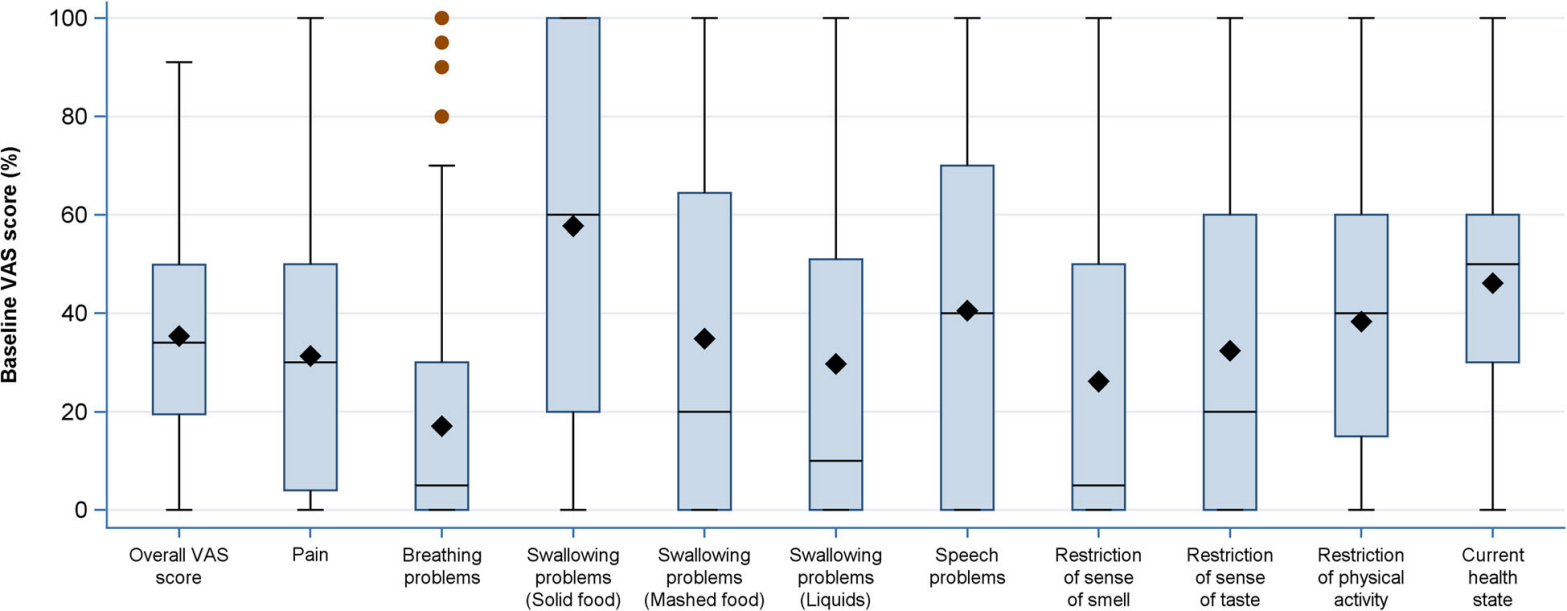
Baseline symptom burden. Baseline symptom burden of the 315 mTAS patients. Values range from 0 to 100, higher values represent heavier symptoms. The point ♦ in the box indicates the mean and the horizontal lines the median

Baseline symptoms of the 315 evaluable patients (mTAS) are given in Fig. 2. The mean overall VAS score before treatment was 35.4, slightly worse than the mean score for pain with 31.3. Most severe symptoms at baseline were swallowing problems with solid food (mean 57.7), followed by speech problems (mean 40.5), and restriction of physical activities (mean 38.3). The self-assessed mean actual overall heath state was 46.1 and thus worse than most of the single symptoms. In addition, baseline symptoms were analyzed separately in patients with locoregional recurrence only (without distant metastases) and in patients with distant metastases (or both). Patients with locoregional recurrence had worse baseline swallowing fuction of solid/ mashed food and liquids and more speech problems compared to patients with distant metastases (supplementary Fig. S3).

### Correlation of treatment response and tumor symptoms

Changes in the patients’ symptom burden are studied for responders (CR/PR) versus non-responders (SD, PD, NA). All changes are displayed for the three time points: The “first post-baseline” assessment compares the first assessment during treatment with the baseline values. The “best post-baseline” assessment compares the best post baseline values of any questionnaire during treatment with the baseline values. The “end of treatment” assessment compares the values at treatment termination with the baseline values. Negative values indicate improved symptoms and positive values deteriorated symptoms.

**Fig. 3:**
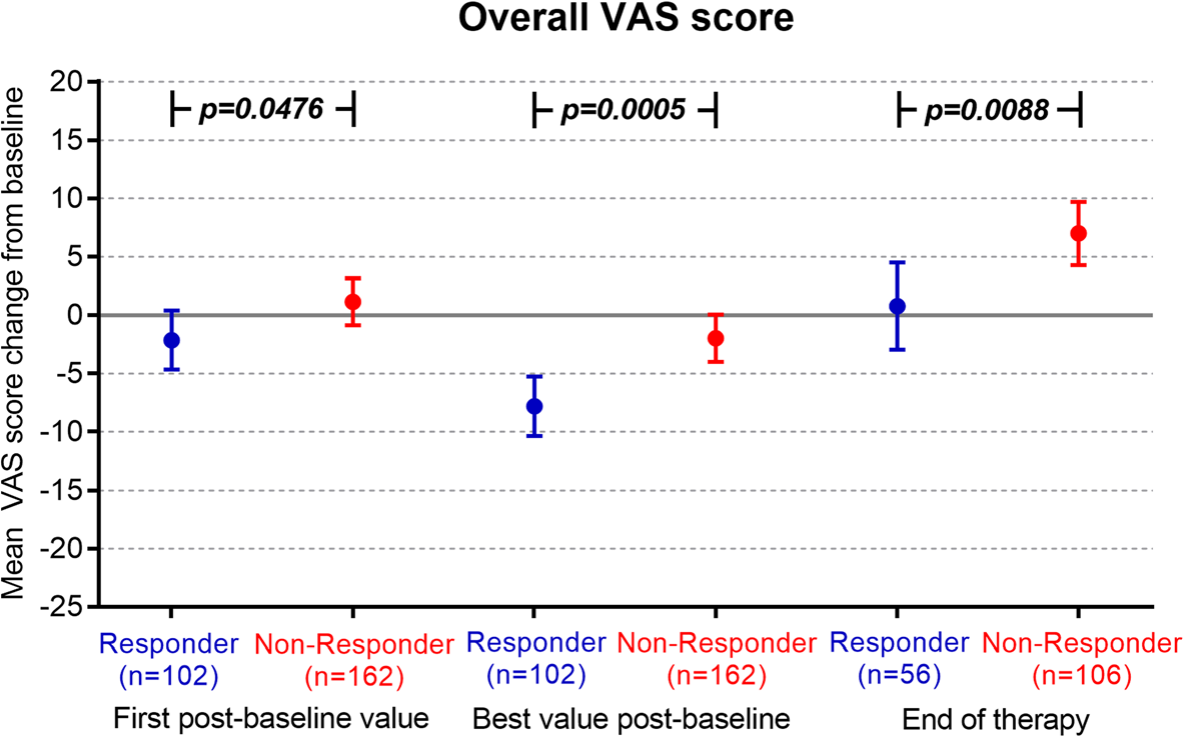
Changes in overall symptom burden in responders and non-responders (ANCOVA analysis). Changes from baseline were analyzed at the three time points “first post-baseline assessment”, “best post-baseline assessment” and “assessment at treatment end” in responders and non-responders. Negative values indicate improved symptoms and positive values deteriorated symptoms. n indicates the number of analyzed questionnaires. Results show the overall VAS score calculated from the ten single symptom VAS scores

The change of overall VAS score from baseline was significantly better in responders compared to non-responders at the first post-baseline assessment (LSM responders − 2.13 vs. non-responders + 1.15, *p* = 0.0476) (Fig. 3). This effect became stronger, when the best post-baseline assessment was chosen (LSM responders − 7.82 vs. non-responders − 1.97, *p* = 0.0005). At end of therapy the mean overall tumor symptom score returned to baseline in responders and deteriorated in non-responders (LSM responders + 0.78 vs. non-responders + 6.99, *p* = 0.0088).

An additional analysis of changes of the overall VAS score in patients with locoregional recurrence only (without distant metastases) and in patients with distant metastases (or both) was performed. Changes of the overall VAS score at the three time points “first post-baseline assessment”, “best post-baseline assessment” and “assessment at treatment end” were similar to the changes in the entire cohort and did not differ in patients with locoregional recurrence and distant metastases (supplementary Fig. S4).

**Fig. 4:**
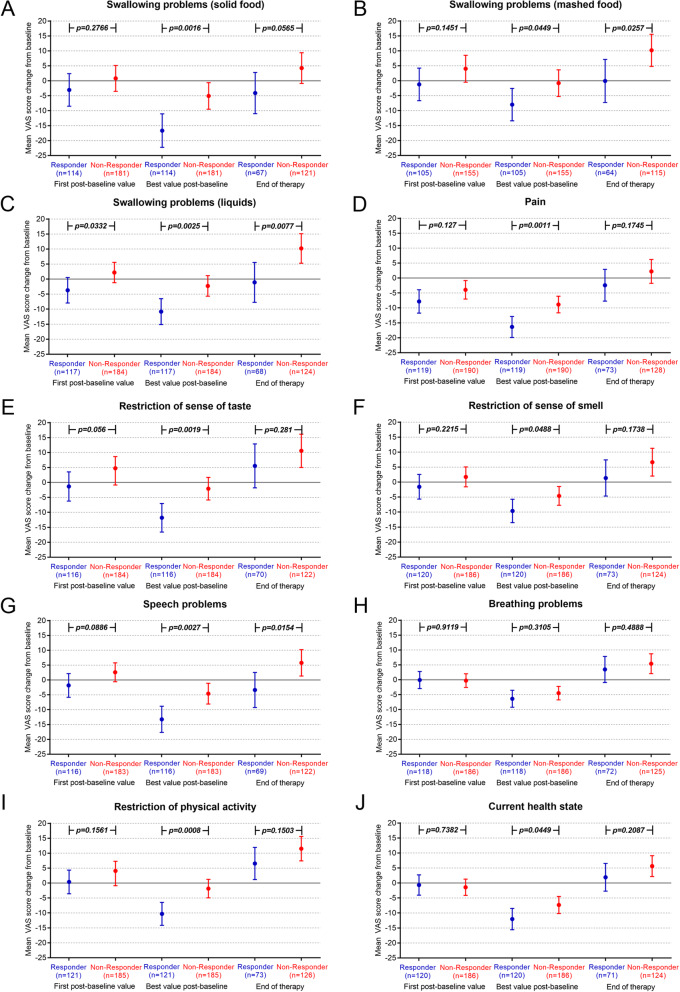
Changes in single tumour symptoms in responders and non-responders (ANCOVA analysis). Changes from baseline were analyzed at the three time points “first post-baseline assessment”, “best post-baseline assessment” and “assessment at treatment end” in responders and non-responders. Negative values indicate improved symptoms and positive values deteriorated symptoms. n indicates the number of analyzed questionnaires. The ten single symptom VAS assessed swallowing of solid food (**a**), swallowing of mashed food (**b**), swallowing of liquids (**c**), pain (**d**), restriction of sense of taste (**e**) and smell (**f**), speech problems (**g**), breathing problems (**h**), restriction of physical activity (**i**) and the self-reported current health state (**j**)

The results of the ten single symptom sub-VAS scores are presented in Fig. 4. In the swallowing assessment, especially solid food was a problem for the patients. At the best post-baseline assessment swallowing of solid food improved significantly stronger in responders (LSM − 16.67 vs. non-responders − 5.06, *p* = 0.0016) (Fig. 4a). For swallowing mashed or liquid food also significant differences in favor of responders were observed at the best post-baseline and the end of therapy assessment (Fig. 4b, c). Larger differences were seen for the symptom pain (Fig. 4d). At the best post-baseline assessment the mean pain score has improved considerably more in responders than in non-responders (LSM: responders − 16.37 vs. non-responders − 8.89, *p* = 0.0011). Similar to swallowing problems, also restriction of smell or taste both were significantly better in responders when the best post-baseline assessment was compared (Fig. 4 e, f). Also speech problems were a main impairment of patients. Speech problems significantly improved more in responders at the best post-baseline assessment (responders − 13.25 vs. non-responders − 4.60, *p* = 0.0027) and remained better until end of treatment (responders − 3.38 vs. non-responders + 5.78, *p* = 0.0154) (Fig. 4g). Responders and non-responders reported no significant differences in breathing problems (Fig. 4h). Responders also evaluated their physical activity and current health state better than non-responders in the best post-baseline assessment (Fig. 4i, j).

Time from treatment initiation is also an important factor. Changes from baseline of the overall VAS score and the single symptoms are reported in monthly intervals (supplementary Fig. S5). Whereas the overall VAS score continuously improves during the first three months in responders, it clearly worsens in non-responders especially at the third month and later. Especially swallowing function of mashed food and liquids slowly improves in responders, whereas it dramatically worsens in non-responders after three months. Physical activity remains stable in responders, whereas it worsens in non-responders already after two months and later.

### Association between baseline factors and OS

In the univariate cox regression analysis to study potentially prognostic factors on OS, older age (especially those between 66 to 75 years), a Charlson score of 0, lower ECOG scores, female sex, and a less severe overall VAS score were associated with lower mortality risk considering all variables with an effect *p*-value of < 0.2 (Table 2). However, alcohol consumption, body weight, type of therapy (RT only, CT only, RCT), duration since initial diagnosis, location of primary tumor (oropharynx, hypopharynx, larynx, mouth/ lip/ tongue, multiple locations, other), type of relapse (loco-regional only, any distant metastases) and smoking status (non-smoker, former smoker, current smoker) were not associated at a p-level of 0.2.

**Table 2:** Univariate and multivariate Cox proportional hazard models to investigate the association between patient characteristics and overall survival

Explanatory factors (TAS cohort, ***N = 391***)		N	Death	HR	95% CI	***p***-value
**Univariate**						
Age at study inclusion*	≤ 65 years	241	153	1		–
	> 65–75 years	107	60	0.68	0.50–0.92	0.0126
	> 75 years	43	28	0.94	0.62–1.38	0.7636
Sex*	Male	320	203	1		–
	Female	71	38	0.66	0.46–0.92	0.0193
Weight	Per 5 kg	391	241	1.02	0.98–1.07	0.3531
Alcohol consumption	Never	87	61	1		–
	Several times per month	85	51	0.9	0.62–1.30	0.5689
	Several times per week or daily	101	60	0.82	0.57–1.17	0.2731
	Missing	118	69	0.74	0.52–1.04	0.0836
Smoking habits	Never smoked	102	60	1		–
	Former smoker	148	94	1.16	0.84–1.61	0.3666
	Current smoker	140	86	1.01	0.73–1.41	0.9393
Charlson comorbidity index at study inclusion*	0	188	110	1		–
	1	79	57	1.37	0.99–1.87	0.0559
	> 1	124	74	1.19	0.88–1.59	0.2545
Chemotherapy/Radiotherapy	Radiotherapy only	78	43	1		–
	Chemotherapy only	309	195	1.17	0.85–1.64	0.3616
	Radio-chemotherapy	4	3	1.82	0.44–5.00	0.3187
Duration since initial diagnosis	Per month	391	241	1	1.00–1.00	0.515
ECOG performance status at treatment initiation*	0	65	31	1		–
	1	225	145	1.63	1.12–2.45	0.0133
	≥ 2	77	53	2.37	1.53–3.73	0.0001
	Unknown	24	12	1.5	0.74–2.86	0.2309
Location of primary tumor	Oropharynx	93	56	1		–
	Hypopharynx	74	45	1.26	0.84–1.86	0.2546
	Larynx	42	26	1.25	0.77–1.96	0.3539
	Oral cavity	89	59	1.18	0.82–1.71	0.3671
	Other	63	35	1.01	0.66–1.53	0.961
	Multiple locations	30	20	1.91	1.12–3.14	0.0134
Disease progression at study inclusion	Local recurrence only	198	125	1		–
	Distant metastases	193	116	0.86	0.66–1.10	0.2276
Overall VAS score*	Per 10 points	276	166	1.12	1.05–1.20	0.0009
**Multivariate**^**a**^						
Overall VAS score	Per 10 points	276	166	1.12	1.05–1.20	0.0009

*TAS* Target Analysis Set, *HR* Hazard Ratio, *CI* Confidence Interval, *ECOG* Eastern Cooperative Oncology Group, *VAS* Visual Analogue Scale. ^a^Final Cox regression model after backward selection. Within the selection process, all explanatory factors with an effect *p*-value of < 0.2 in the univariate Cox regression analysis were considered (*). Only factors with *p* < 0.05 remained in the final model

In the multivariable analysis only the overall VAS score remained a prognostic factor for overall survival, with hazard increase of 12% per 10 points increment for the overall VAS score at baseline. (hazard ratio: 1.12 per 10 points in VAS, 95% CI 1.05–1.20, *p* = 0.0009) (Table 2).

## Discussion

The SOCCER trial is a prospective multi-center non-interventional trial in patients with recurrent and/or metastatic HNSCC treated with cetuximab-chemotherapy or cetuximab-radiotherapy combination. The trial showed a clear association between treatment response and reduced tumor-related symptoms. However, in the current era of checkpoint inhibitors the study endpoint treatment response lost importance. In the phase III first-line study comparing pembrolizumab with platinum/5-flurouracil/cetuximab (Keynote-048), PD-L1 positive patients (combined positive score ≥ 1) had a significantly higher OS in the pembrolizumab-arm, despite a much lower response rate of 19% compared to 35% in the EXTREME-arm [4]. Nevertheless, good palliative treatment should not only prolong survival, but also improve the patients’ symptoms and consequently their quality of life (QoL). Thus, treatment regimens with high response rates as chemotherapy may be more efficient in controlling tumor symptoms than regimens with low response rates as single agent immunotherapiy. Two phase III trials in recurrent and/or metastatic HNSCC also addressed patients’ QoL using the EORTC QLQ-C30 and QLQ-H&N35 questionaires. In the randomized phase III trial comparing nivolumab with investigators choice in a second line setting (CheckMate-141), QoL did not change in the nivolumab arm, whereas it became worse in the investigators choice arm [6]. However, the first line platinum/5-flurouracil/cetuximab combination showed a significant improvement in QoL in the EXTREME trial [7].

It may be a limitation of the relevance of our results that an improvement of tumor symptoms is generally expected after treatment response by experienced clinicians. However, the SOCCER trial quantified the effect of the improvement and displayed the effect on the most important symptoms. This provides data for clinical decisions, which symptoms may be treated by intense tumor-directed treatments instead of supportive care only. Compared to the trials mentioned above, the questionnaire of the SOCCER trial was study specific and not evaluated before. Thus, no comparison of the results with other trials using evaluated quality of life assessments is possible, which is a limitation of the trial. The questionnaire in the SOCCER trial was designed to evaluate the effect of treatment response on specific tumor symptoms in a short questionnaire. As it was not the aim of the trial to assess quality of life, no information was gathered for typical quality of life items as role, social, emotional or cognitive functioning. As these factors were not assessed, it can be speculated that found symptom improvements in responders in the SOCCER trial might have been biased. Patients who knew they are responding might have answered symptom questions more positively. This speculation can be cleared as tumor symptoms (overall VAS) already significantly improved in the first post baseline assessment, which was performed long before the first tumor staging procedure.

A unique feature of the current SOCCER trial is the large multicenter prospective cohort displaying unselected patient data as a relevant number of patients had ECOG 2 and a Charlson comorbidity index ≥1, who were not included in the phase III trials mentioned above. Furthermore, the received rate of pairs of VAS questionnaires of 67.0% was much higher than in previous trials, e.g. in the nivolumab second line trial with only 39% or the EXTREME trial with 44% (regarding EORTC QLQ-C30) [6, 7]. A limitation of this trial is that the cohort mainly contains patients treated with cetuximab in combination with platinum-based chemotherapy, but also fewer patients treated with radiotherapy and cetuximab. The overall response rate in this trial was 33%, which is comparable to previously reported 36% of the EXTREME trial [1]. However, in current phase III trials with a cisplatin/5-flurouracil/cetuximab in the control arm the overall response rates might be slightly higher. This combination achieved an overall response rate of 36% in the Keynote-048 trial [4] and 40% in the TPExtreme trial [8].

As mentioned above, treatment response improved tumor related symptoms in first line treatment with cetuximab-chemotherapy or cetuximab-radiotherapy combination. This effect appeared fast, as the improvement was visible in the first post-baseline assessment scheduled after four weeks. At the end of treatment, most transiently improved tumor symptoms returned to baseline in the responders and became worse in the non-responders, which is probably an effect of tumor progression. Treatment response improved several different symptoms of the patients like swallowing of solid food, mashed food and liquids. The responders also reported improved senses of smell and taste. Taking into account, that some of these symptoms might also be caused by prior surgery and/or chemoradiation [9], patients with tumor induced symptoms might have even a greater benefit. Especially for these patients with tumor induced swallowing problems, treatment schemes with high response rates should be preferred. Another obvious improvement was seen in pain. Systemic treatment schemes with high response rates might have a superior effect on pain than only administrating analgesics. Furthermore, responders also had less speech problems than non-responders. Interestingly, in the EXTREME trial the greatest benefits were also found in swallowing, pain and speech [7].

These findings are of high relevance for clinical treatment algorithms. As mentioned above, PD-1 inhibitors changed treatment algorithms in recurrent and/or metastatic HNSCC as pembrolizumab was approved for first line treatment either alone or in combination with chemotherapy. Most checkpoint-inhibitor trials suggest a clinical treatment algorithm only based on the PD-L1 status. The SOCCER trial highlights the relevance of treatment response to improve tumor symptom burden, especially dysphagia and pain. A clinical consequence of SOCCER might be to consider treatments with low response rates as PD-1 inhibitor monotherapy more for patients with lower symptom burden and and treatment regimens with higher response rates as chemotherapy-containing combinations for patients with more severe tumor symptom burden. High response rates of around 36% can be induced by platinum/5-flurouracil either in combination with cetuximab or pembrolizumab [4] or with the combination of cetuximab and radiotherapy as presented here. For the cisplatin/docetaxel/cetuximab combination even response rates up to 46% have been reported [8]. Another highly effective option for patients with loco-regional relapse can also be reirradiation in combination with cetuximab [10]. This combination achieved an overall response rate of 74% in the phase III trial (“Bonner trial”) in treatment-naive patients and was used in a sub-cohort in the current SOCCER trial (20% of included patients).

A further finding of this non-interventional study is that severe tumor symptom burden correlates with reduced overall survival. The risk for death increases by 12% per 10 points of the overall VAS score (hazard ratio 1.12). This is not surprising as swallowing problems may lead to aspiration and subsequent pneumonia or breathing problems may lead to hypoxia. These are potentially life-threatening situations. This supports the idea to use platinum-based regimens with higher response rates in patients with severe tumor symptoms instead of regimens with lower response rates as single agent immunotherapy. In patients with loco-regional relapse also reirradiation in combination with cetuximab should be considered. Nevertheless, these are intensive treatments with relevant toxicity. It must be a future aim to develop treatment regimens with high response rates and low toxicity especially for palliative situations.

## Conclusions

Taken together, in unselected patients beyond randomized controlled trials, treatment response lowers tumor symptom burden in recurrent and/or metastatic HNSCC. Tumor symptom burden detected by this 10-item VAS questionnaire was an independent prognostic value for overall survival.

## Supplementary information

**Additional file 1: Supplementary Fig. S1.** Visual analogue scale (VAS) questionnaire.

**Additional file 2: Supplementary Fig. S2.** Baseline symptoms in patients without and with distant metastases

**Additional file 3: Supplementary Fig. S3.** Changes in overall symptom burden in responders and non-responders without and with distant metastases. (PDF 280 kb)

**Additional file 4: Supplementary Fig. S4.** Survival analyses according to the Kaplan-Meier Method.

**Additional file 5: Supplementary Fig. S5.** Time-dependence of tumor symptom burden in responders and non-responders.

## Data Availability

The data that support the findings of this study are available from the corresponding author upon reasonable request after permission of the funding company Merck Serono GmbH (an affiliate of Merck KGaA, Darmstadt, Germany).

## Abbreviations

ANCOVA: Analysis of covariance
CI: Confidence interval
CR: Complete response
DCR: Disease control rate
ECOG: Eastern Cooperative Oncology Group
EMA: European Medicines Agency
HNSCC: Head and neck squamous cell cancer
HR: Hazard ratio
mTAS: Modified Target Analysis Set
NA: Not assessable
ORR: Overall response rate
OS: Overall survival
PD: Progressive disease
PD-1: Programmed cell death 1 protein
PD-L1: Programmed cell death ligand 1
PFS: Progression-free survival
PR: Partial response
QoL: Quality of life
SD: Stable disease
TAS: Target Analysis Set
VAS: Visual analogue scale
