# Supplementary figures and images for: Treatment response lowers tumor symptom burden in recurrent and/or metastatic head and neck cancer
Source: BMC Cancer. 2020 Sep 29;20:933. doi: 10.1186/s12885-020-07440-w (PMC7526421; doi:10.1186/s12885-020-07440-w)

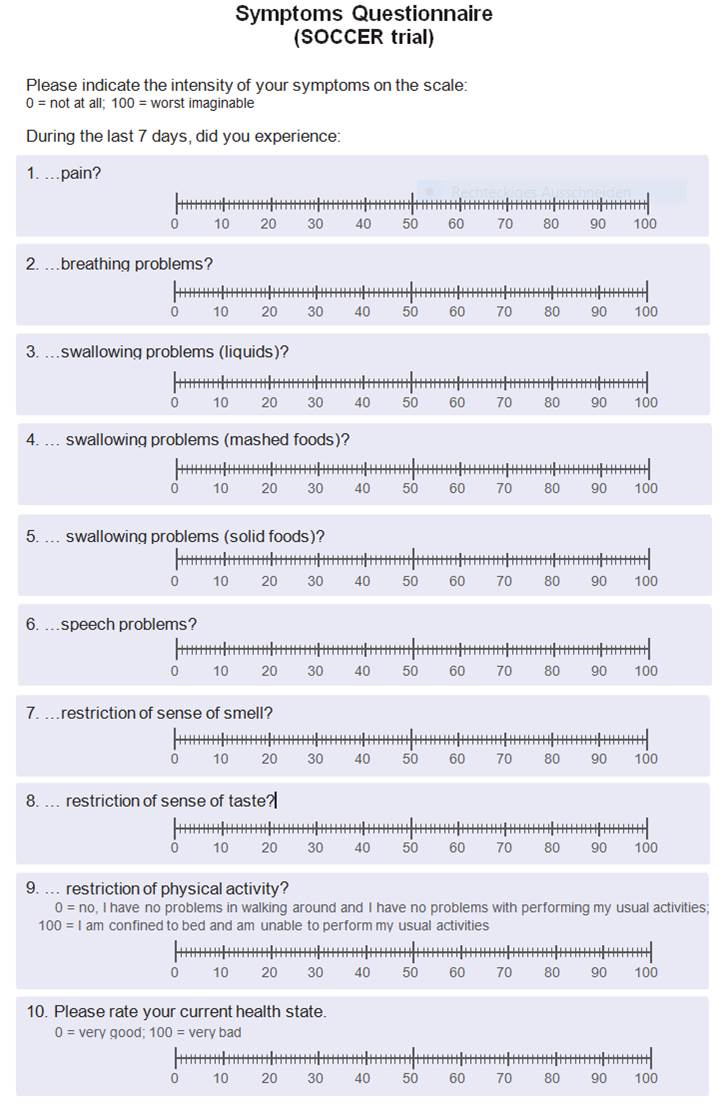

Supplement: Supplementary file 1 — Additional file 1: Supplementary Fig. S1. Visual analogue scale (VAS) questionnaire. [file 12885_2020_7440_MOESM1_ESM.tif]
