# Supplementary material for: Treatment response lowers tumor symptom burden in recurrent and/or metastatic head and neck cancer
Source: BMC Cancer. 2020 Sep 29;20:933. doi: 10.1186/s12885-020-07440-w (PMC7526421; doi:10.1186/s12885-020-07440-w)
Supplement: Supplementary file 2 — Additional file 2: Supplementary Fig. S2. Baseline symptoms in patients without and with distant metastases [file 12885_2020_7440_MOESM2_ESM.pdf]

Supplementary Figure S2:

Baseline symptoms in patients without and with distant metastases

A)

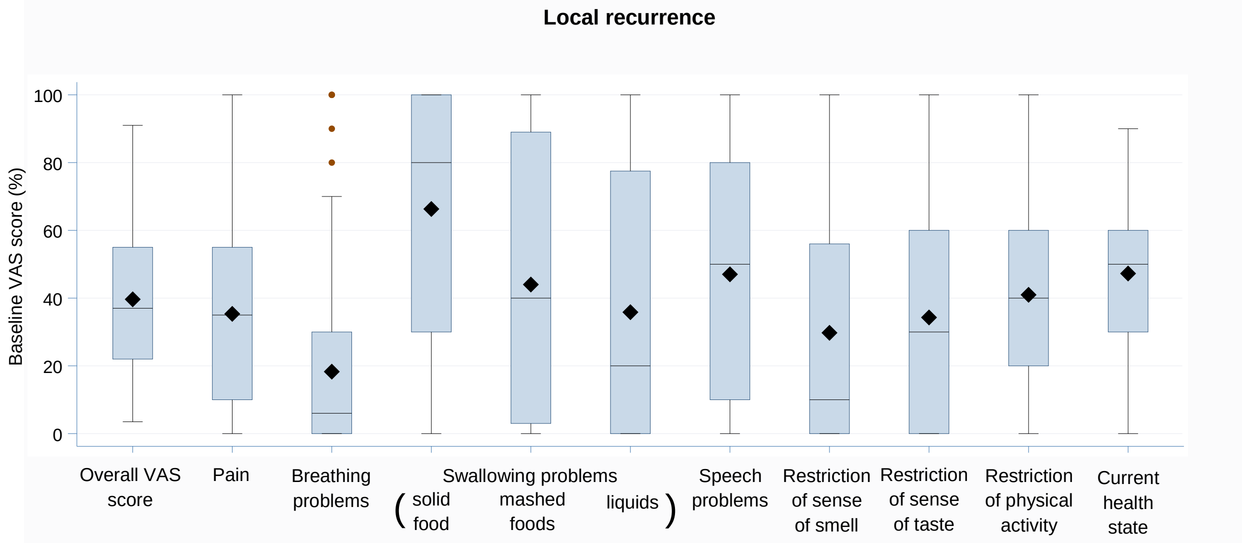

B)

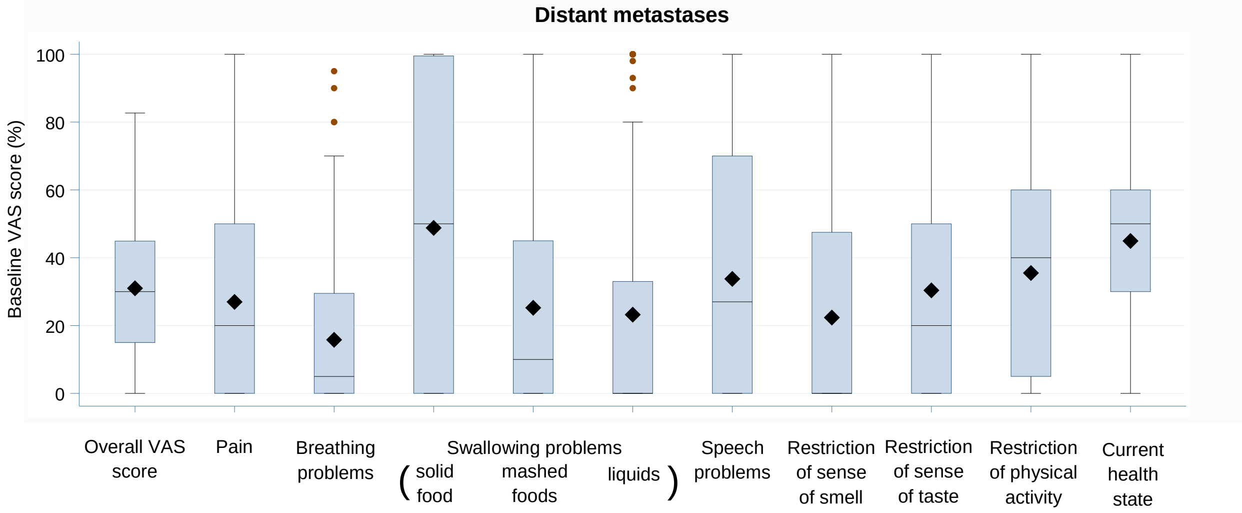

Baseline symptom burden of the mTAS patients separated in patients with locoregional recurrence without distant metastases (A) and patients with distant metastases or both (B). Values range from 0 – 100, higher values represent heavier symptoms. The point ♦ in the box indicates the mean and the horizontal lines the median.
