# Supplementary material for: Treatment response lowers tumor symptom burden in recurrent and/or metastatic head and neck cancer
Source: BMC Cancer. 2020 Sep 29;20:933. doi: 10.1186/s12885-020-07440-w (PMC7526421; doi:10.1186/s12885-020-07440-w)
Supplement: Supplementary file 3 — Additional file 3: Supplementary Fig. S3. Changes in overall symptom burden in responders and non-responders without and with distant metastases. (PDF 280 kb) [file 12885_2020_7440_MOESM3_ESM.pdf]

Supplementary Figure S3:

Changes in overall symptom burden in responders and non-responders without and with distant metastases (ANCOVA analysis)

A)

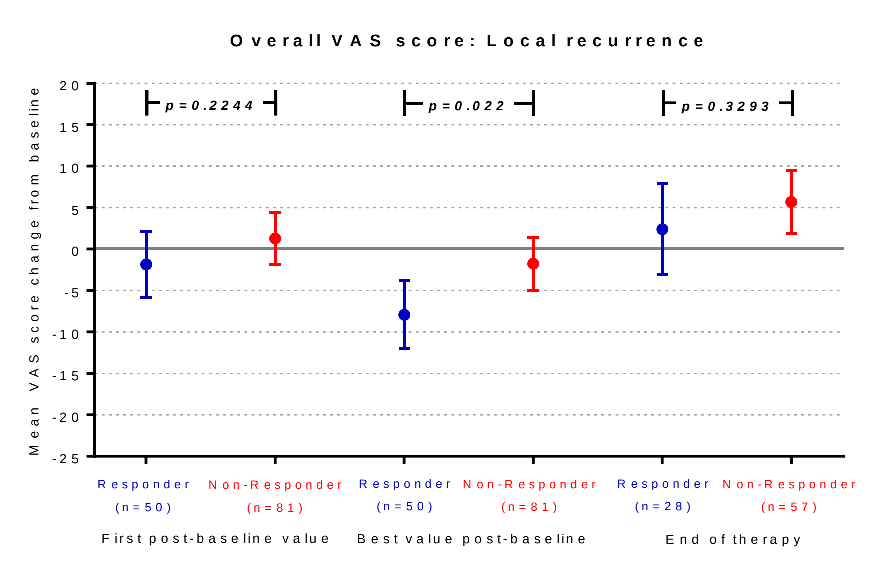

B)

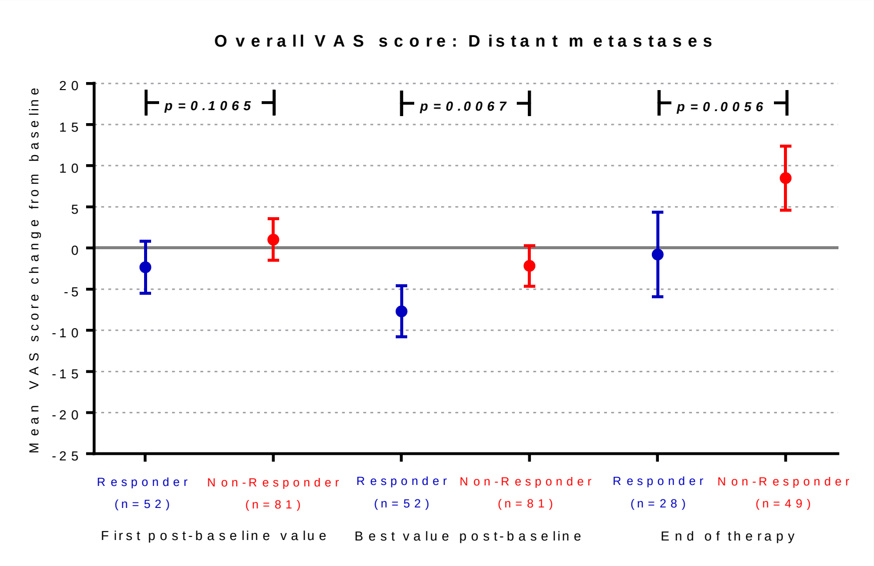

Changes from baseline in the patients' overall symptom burden were analyzed in patients with locoregional recurrence without distant metastases (A) and patients with distant metastases or both (B). The three time points "first post-baseline assessment", "best post-baseline assessment" and "assessment at treatment end" were analyzed in responders and non-responders. Negative values indicate improved symptoms and positive values deteriorated symptoms. n indicates the number of analyzed questionnaires. Results show the overall VAS score calculated from the ten single symptom VAS scores.
