# Supplementary material for: Treatment response lowers tumor symptom burden in recurrent and/or metastatic head and neck cancer
Source: BMC Cancer. 2020 Sep 29;20:933. doi: 10.1186/s12885-020-07440-w (PMC7526421; doi:10.1186/s12885-020-07440-w)
Supplement: Supplementary file 4 — Additional file 4: Supplementary Fig. S4. Survival analyses according to the Kaplan-Meier Method. [file 12885_2020_7440_MOESM4_ESM.pdf]

Supplementary Figure S4:

Survival analyses according to the Kaplan-Meier Method.

A

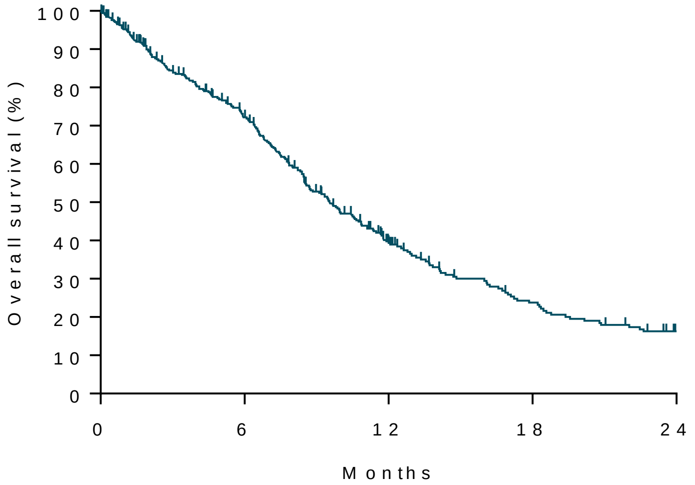

Number at risk

Total population	389	228	98	45	19

B

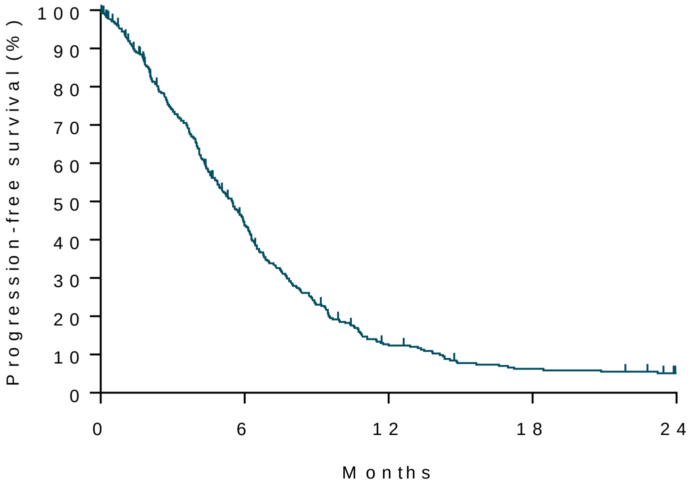

Number at risk

Total population	388	142	38	17	7

Kaplan-Meier curves for overall survival (A) and progression-free survival (B) for all patients (TAS cohort). One patient was excluded from the PFS analysis due to missing follow up.
