# Supplementary material for: Treatment response lowers tumor symptom burden in recurrent and/or metastatic head and neck cancer
Source: BMC Cancer. 2020 Sep 29;20:933. doi: 10.1186/s12885-020-07440-w (PMC7526421; doi:10.1186/s12885-020-07440-w)
Supplement: Supplementary file 5 — Additional file 5: Supplementary Fig. S5. Time-dependence of tumor symptom burden in responders and non-responders. [file 12885_2020_7440_MOESM5_ESM.pdf]

Supplementary Figure S5:

Time-dependence of tumor symptom burden in responders and non-responders.

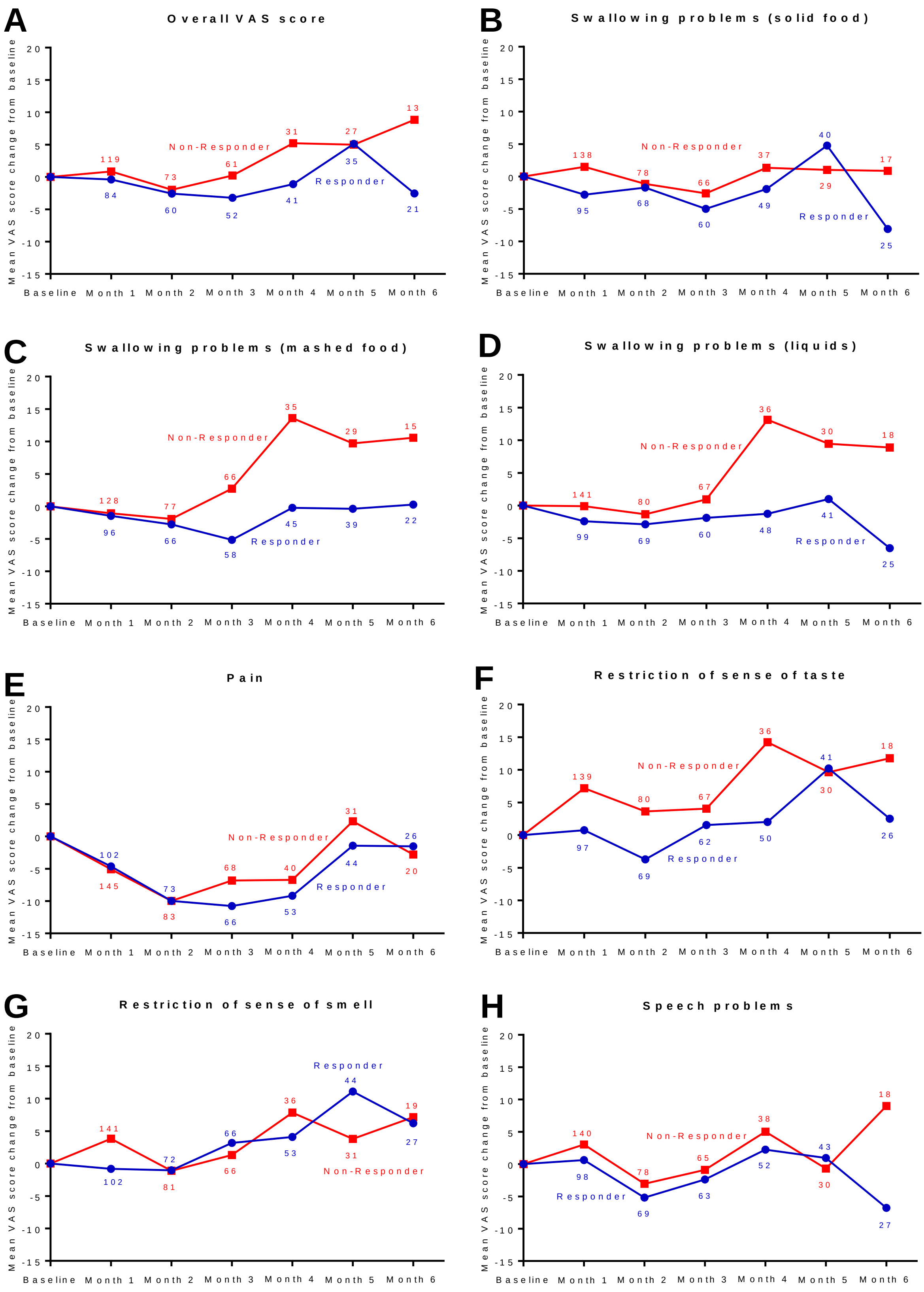

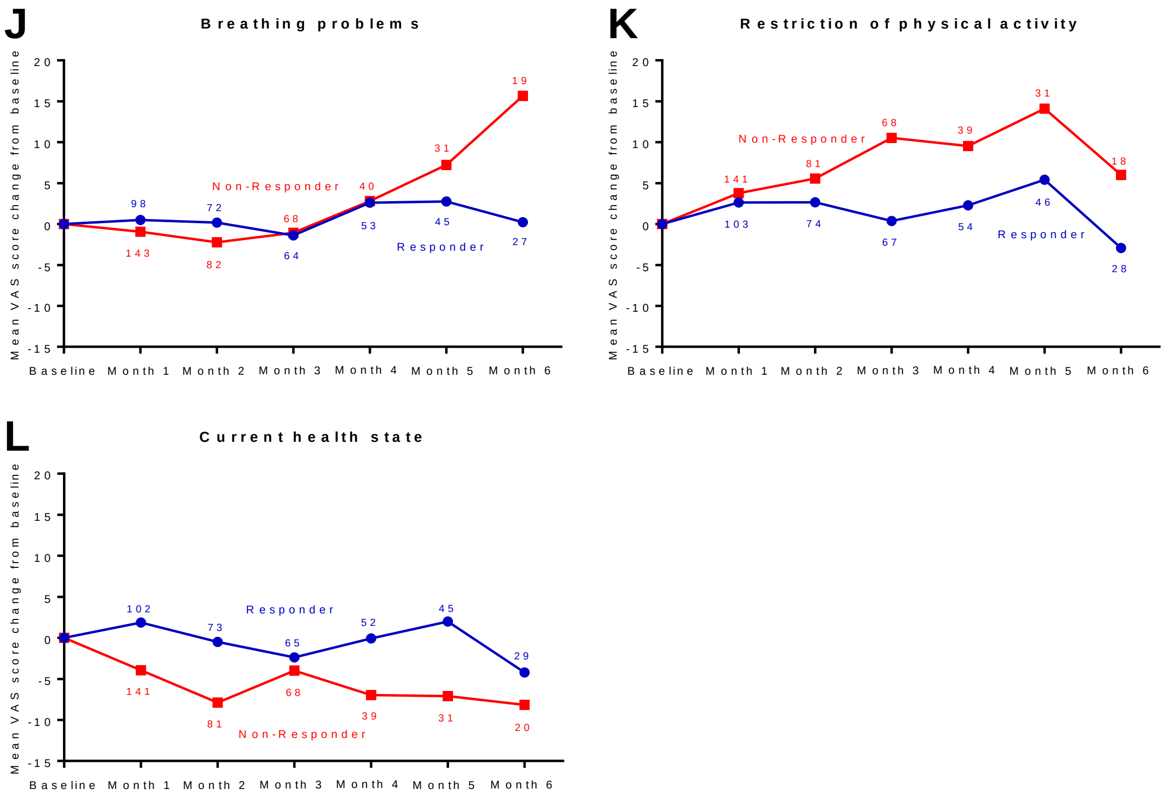

Changes of the patients' tumor symptom burden from baseline were analyzed every month after treatment initiation. Negative values indicate improved symptoms and positive values deteriorated symptoms. The number of analyzed questionnaires is given above or below the points. The overall VAS score is given in (A). The ten single symptom VAS assessed swallowing of solid food (B), swallowing of mashed food (C), swallowing of liquids (D), pain (E), restriction of sense of taste (F) and smell (G), speech problems (H), breathing problems (J), restriction of physical activity (K) and the self-reported current health state (L).
